# An efficient method for generating a germ cell depleted animal model for studies related to spermatogonial stem cell transplantation

**DOI:** 10.1186/s13287-016-0405-1

**Published:** 2016-09-22

**Authors:** Nirmalya Ganguli, Neerja Wadhwa, Abul Usmani, Neetu Kunj, Nilanjana Ganguli, Rajesh Kumar Sarkar, Soma M. Ghorai, Subeer S. Majumdar

**Affiliations:** 1 Embryo Biotechnology Laboratory, National Institute of Immunology, Aruna Asaf Ali Marg, New Delhi, 110067 India; 2 Department of Zoology, University of Delhi, Delhi, 110 007 India; 3 National Institute of Animal Biotechnology, Hyderabad, Telengana India

**Keywords:** Busulfan, Germ cell depletion, Germ cell transplantation, Fertility restoration, Chemotherapy

## Abstract

**Background:**

Spermatogonial stem cell (SSC) transplantation (SSCT) has become important for conservation of endangered species, transgenesis and for rejuvenating testes which have lost germ cells (Gc) due to gonadotoxic chemotherapy or radiotherapy during the prepubertal phase of life. Creating a germ cell-depleted animal model for transplantation of normal or gene-transfected SSC is a prerequisite for such experimental studies. Traditionally used intraperitoneal injections of busulfan to achieve this are associated with painful hematopoietic toxicity and affects the wellbeing of the animals. Use of testicular busulfan has been reported recently to avoid this but with a very low success rate of SSCT. Therefore, it is necessary to establish a more efficient method to achieve higher SSCT without any suffering or mortality of the animals.

**Methods:**

A solution of busulfan, ranging from 25 μg/20 μl to 100 μg/20 μl in 50 % DMSO was used for this study. Each testis received two diagonally opposite injections of 10 μl each. Only DMSO was used as control. Germ cell depletion was checked every 15 days. GFP-expressing SSC from transgenic donor mice C57BL/6-Tg (UBC-GFP) 30Scha/J were transplanted into busulfan-treated testis. Two months after SSCT, mice were analyzed for presence of colonies of donor-derived SSC and their ability to generate offspring.

**Results:**

A dose of 75 μg of busulfan resulted in reduction of testis size and depletion of the majority of Gc of testis in all mice within 15 days post injection without causing mortality or a cytotoxic effect in other organs. Two months after SSCT, colonies of donor-derived Gc-expressing GFP were observed in recipient testes. When cohabitated with females, donor-derived offspring were obtained. By our method, 71 % of transplanted males sired transgenic progeny as opposed to 5.5 % by previously described procedures. About 56 % of progeny born were transgenic by our method as opposed to 1.2 % by the previously reported methods.

**Conclusions:**

We have established an efficient method of generating a germ cell-depleted animal model by using a lower dose of busulfan, injected through two diagonally opposite sites in the testis, which allows efficient colonization of transplanted SSC resulting in a remarkably higher proportion of donor-derived offspring generation.

**Electronic supplementary material:**

The online version of this article (doi:10.1186/s13287-016-0405-1) contains supplementary material, which is available to authorized users.

## Background

Fertility of males in mammalian species depends upon the production of sperm through the process of spermatogenesis. Spermatogonial stem cells (SSC) are the male germline stem cells which stand to be the foundation of spermatogenesis. SSC are present on the basal membrane of the seminiferous tubules in the testis and surrounded by Sertoli cells, which function as an important component of the SSC niche [1]. SSC differentiate and divide to form the mature spermatozoa. In 1994, Brinster and Zimmerman first demonstrated the possibilities of isolation and transplantation of SSC into an evacuated testis followed by successful spermatogenesis from the transplanted SSC [2]. Since then, this approach has been explored to address various issues like transgenesis [3, 4], infertility [5], conservation of endangered species [6], and for understanding the biology of male germinal stem cells and its niche [3], etc. Fertility preservation is an important issue in the management of the quality of life of prepubertal boys undergoing gonadotoxic cancer therapy because sperm cryopreservation is not possible for them due to sexual immaturity at that phase of development [7]. Presently, the only theoretical option for preservation of fertility in these boys is the preservation of the SSC for intratesticular stem cell transplantation [8, 9]. The major requirement for successful germ cell transplantation (GCT) is the preparation of germ cell-depleted recipients (GCD) in which endogenous Gc are destroyed, creating space for the transplantation of exogenous SSC [10, 11]. Various approaches like irradiation [12], heat shock treatment [13], and chemical treatment have been adopted to generate evacuated testis to prepare recipients for GCT. The sole chemical treatment most commonly used for the preparation of recipients is through intraperitoneal (i.p.) injection of busulfan [14] due to its easy handling and availability [14]. Busulfan (1,4-butanediol dimethane sulfonate), a DNA alkylating agent is often used to kill endogenous Gc, creating an empty space in the basal and adluminal compartment of seminiferous tubules prior to SSC transplantation [14]. It mediates cytotoxic effects through the formation of DNA-DNA cross-links, DNA-protein cross-links and single-strand breaks. Busulfan exerts its toxic effects on cells that are at the G0/G1 phase of the cell cycle [15, 16]. Intraperitoneal administration of busulfan preferentially kills proliferating cells including spermatogonial stem cells, leading to male infertility [17]. But there are several drawbacks of this existing technique of creating GCD by i.p. injection of busulfan. It takes almost 6 weeks for successful depletion of Gc. Busulfan doses, less than 40 mg/kg given to adult mice did not result in prolonged depletion of endogenous spermatogenesis in most tubules [14]; higher doses often caused severe hematopoietic suppression requiring bone marrow transplantation and resulted in death [18]. Therefore it was hypothesized that after i.p. injection, busulfan may be absorbed and transferred to other organs via blood circulation and exert a nonspecific cytotoxic effect on the body by destroying other cellular niches of the body resulting in bone marrow depression and anemia.

To overcome these drawbacks, an alternative method of testicular busulfan injection was explored in sheep [19] where busulfan was directly injected in the scrotal region, but transplantation experiments were not carried out in this species. Recently, the testicular route was explored in rodents [20, 21], but is much less efficient. Only 4 out of 335 pups originated from donor SSC which were transplanted exogenously in mouse testes treated directly with 120–180 μg of busulfan [21]. Only 1 out of 18 recipient mice transplanted with SSC sired 4 transgenic pups, displaying a success rate of 5.5 % [21]. Therefore, the aim of the present study was to explore a method to achieve more Gc depletion and a relatively higher success rate of Gc transplantation to increase efficiency of direct testicular busulfan treatment. The success rate of GCT is directly correlated to the efficiency of generating donor Gc-derived offspring.

Here, we have established a method of testicular busulfan injection which requires lower dose of busulfan to prepare recipients for GCT while achieving the objective of maximum Gc depletion without any cytotoxic effect in other organs. The success rate by our method is 56 % which is about severalfold more as compared to 1.2 % shown earlier [21]. This method is fast, efficient and ethically superior as it will utilize fewer animals to generate more information.

## Methods

### Animals

Six-week-old F1Bl6SJL (F1 hybrid of Bl6 and SJL) and FVB mice were used for busulfan treatment and as recipients for SSC transplantation. Six-week-old GFP-expressing transgenic C57BL/6-Tg (UBC-GFP)30Scha/J male mice were used as donors of testicular Gc. All mice were bred and maintained at the Small Animal Facility of the National Institute of Immunology. All mice were kept at 24 ± 2 °C under a 14 hours light and 10 hours dark cycle and used as per the National Guidelines provided by the Committee for the Purpose of Control and Supervision of the Experiments on Animals (CPCSEA). Protocols for the experiments were approved by the Institutional Animal Ethics Committee.

### Busulfan treatment (BST)

Busulfan (Sigma-Aldrich, St Louis, MO, USA) was dissolved in dimethyl sulfoxide (DMSO, Sigma-Aldrich), and then an equal volume of sterile water was added to obtain an aqueous solution of busulfan. This was maintained at 42 °C until injection, with frequent vortexing, to prevent the busulfan from crystallizing. Six-week-old recipient male mice (body weight approximately 30 g) were randomly distributed into various testicular busulfan treatment groups (T-BST).The mice were injected with various doses of busulfan, i.e., 25 μg (T-BST-25) or 50 μg (T-BST-50) or 75 μg (T-BST-75) or 100 μg (T-BST-100) delivered in a total volume of 20 μl through two different injection sites (approximately 10 μl per injection) in each testis. The negative control group received testicular injection of 50 % DMSO in 20 μl distilled water (T-DMSO). The intraperitoneal busulfan treatment group (IP-BST) received injections of 40 mg/kg busulfan [2, 22, 23]. Mice were anesthetized by intraperitoneal injection (200 μl) of ketamine hydrochloride (45 mg/kg) and xylazine hydrochloride (8 mg/kg) before busulfan or DMSO treatment. For testicular injections of busulfan, hair was removed from the lower abdominal and scrotal area of the mice. The area was then wiped with betadine (povidone iodine) followed by 70 % ethanol. The testes were gently squeezed from the abdominal cavity into the scrotum. Busulfan solution containing 0.04 % Trypan blue, which was used to monitor the accuracy of the injection, was injected slowly into the testis using the 10-μl Hamilton syringe (701 N; Hamilton Bonaduz AG, Switzerland). The injection was given at two different and diagonally opposite sites of the testes (10 μl at each site, 20 μl/testis) covering the whole testes (Additional file 1: Figure S1a and b). After injection, the site was again wiped with betadine solution and mice were kept under lamps until consciousness was regained.

### Mortality rate, body weight and testicular weight post BST

After busulfan treatment, the mice were euthanized by cervical dislocation at 15, 30, 60 and 90 days respectively and the body weight of mice from each group was recorded. Testicular size and testicular weight of the mice was measured at day 15 post BST. Mortality rate was observed in busulfan-treated mice.

### Histological analysis

Testes and liver tissue of the BST and control mice were dissected, washed briefly in PBS and fixed in 4 % paraformaldehyde for 18–20 hours. After complete dehydration in a graded series of ethanol and permeabilization in xylene, the tissues were embedded in paraffin, sectioned at 5 μm using Reicher Jung, 2040; Microtome (Leica Biosystems, Nussloch Germany).Tissue histology was performed as described by us previously [24]. Testicular tissue sections were stained with hematoxylin and eosin for histological examination for evaluating the status of spermatogenesis and were observed under bright field illumination with Nikon Eclipse TE2000-S inverted microscope (Nikon Corporation, Tokyo, Japan) attached to a DS-5 M camera assisted by Digital Sight DS-L1 software for capturing the images.

### Routine blood analysis

After 15 days of T-BST-75 injection, peripheral blood from experimental and wild-type control mice (Wt-Ctrl) were collected by retro-orbital bleeding in tubes with EDTA according to Hoff’s method [25]. White blood cells (WBC), red blood cells (RBC), platelets (PLT) and hemoglobin (Hb) along with other hematological parameters were analyzed using an automated analyzer (MS Pharmaceuticals, Amman, Jordan).

### Sperm count and fertility assessment post BST

After 15 days of T-BST-75 injection, the number of epididymal spermatozoa were analyzed by counting total numbers of sperm present in each cauda epididymis after releasing the sperm in 1 ml of 1X PBS by puncturing the epididymis at several sites. The total sperm count was determined using a hemocytometer under a light microscope. For analysis of litter size, after 15 days of busulfan injections three T-BST-75-treated males were cohabitated with age-matched female mice and number of offspring produced was recorded.

### Preparation of donor testicular germ cells

Testicular Gc from the 6-week-old transgenic C57BL/6-Tg(UBC-GFP) 30Scha/J transgenic donor mice were isolated using the procedure adapted from Bellve et al., 1977 and Guan et al., 2006 [26, 27] with little modifications. Briefly, testes obtained post castration of male mice were decapsulated and washed in HBSS before mincing. Minced testicular tissue was washed in HBSS, to eliminate blood cells and non-adherent interstitial cells as supernatant. The sedimented seminiferous tubules were suspended in 25 ml of prewarmed collagenase solution (1200 U collagenase/25 ml HBSS) containing 100 Kunitz units (KU) of deoxyribonuclease (DNase) and the digestion was carried out at 34 °C for 10 minutes in a shaking water bath at 120 oscillations/minute. Ten milliliters of supernatant was carefully aspirated at this point, and kept on ice. Collagenase digestion was resumed for the remaining 15 ml of seminiferous tubules for 15 to 20 minutes, until most of the seminiferous tubules got finely digested. The cell-rich suspension was carefully aspirated and the tissue debris and detritus were discarded. The tissue suspension (approximately 24 ml) was distributed into four 15 ml polypropylene conical tubes, which were centrifuged for 5 minutes at 600 rpm (127 g) at 4 °C. The supernatant was aspirated and centrifuged at 2500 rpm for 5 minutes at 4 °C. The pellet was resuspended in 15 ml HBSS and the cells were dispersed by pipetting. The cell suspension was allowed to stand at 4 °C for 5 minutes, to allow the sedimentation of heavier somatic cells, tissue aggregates, etc. The upper cell suspension was aspirated and the sediment was discarded. Cell suspension was centrifuged at 2500 rpm to obtain a dense cell pellet.

### Antibody staining and FACS-mediated sorting of testicular cells

The dense cell pellet containing crude preparation of Gc was incubated with mouse CD 90.2 antibody (Ab) directly conjugated to PE (phycoerythrin) at 1:100 dilution with sterile 1XPBS for 45 minutes. CD90.2 is a marker for SSC of the testis. A small fraction of cells previously separated were used as a control during sorting. Unbound antibody was removed by washing with cold HBSS and cells were subsequently suspended in filtered DMEM with 1 % serum and filtered through 70-μm filter (BD Biosciences, San Jose, CA, USA). Unstained fraction of cells were likewise washed and filtered. Such stained and unstained cell suspensions were filtered using a 100-μm filter to obtain clump-free cells and further used for FACS-mediated SSC isolation using BD FACS Aria III cell sorter (BD Biosciences, USA). The CD90.2/PE-positive cells were sorted with an 85-μm nozzle at 45 psi sheath pressure and at a flow rate of 3000 events per second in the “4-way purity” mode. The sorted cells were collected in DMEM supplemented with 2 % FCS. Laser 561 was used for excitation of the PE signals.

### Culture, expansion and transplantation of spermatogonial stem cells

SSC selected by FACS using CD90.2 antibody were cultured in vitro [27]on Matrigel-coated plates in a defined growth factor medium. Culture media consisted of Stem-Pro34 SFM medium, 1X Stem-Pro34 supplement, 1X N2 supplement, 1X MEM vitamin, 1X nonessential amino acids, 30 ng/ml β-estradiol, 60 ng/ml progesterone, 1000 U/ml leukemia inhibitory factor (LIF), 10 ng/ml basic fibroblast growth factor (bFGF), 10 ng/ml glial fibriliary-derived nerve growth factor (GDNF), 10 μg/ml insulin, 5 μg/ml transferrin, 5 μg/ml sodium selenite, 2 mM L-glutamine, 6 mg/ml D-(+) glucose, 30 μg/ml pyruvic acid, D-L lactic acid, 5 mg/ml bovine albumin, 50 μM beta mercaptoethanol, 2.5 ng/ml epidermal growth factor (EGF) and 1 % FCS. Cells were maintained at 34 °C and 5 % CO_2_ environment. The cells were maintained in culture for several days to increase their numbers. Fifteen days after testicular busulfan injection, these cells were transplanted into testis of T-BST-75. Briefly, 0.5–1.0 × 10^5^ of cells were suspended in 10 μl PBS along with 0.4 % Trypan blue tracking dye and transplanted into the seminiferous tubules of the testes of recipient mice through the efferent duct injection using a 50-μm glass micropipette controlled by a FemtoJet microinjector. The success of the injection was monitored by observing the distribution of the Trypan blue dye in the convoluted seminiferous tubules. The mice were sacrificed 2 months later, and the testes were examined under a fluorescence stereo zoom microscope to detect GFP expression.

### Analysis of recipient testes after SSC transplantation

Sixty days post transplantation, the testes of the transplanted and non-transplanted T-BST-75 recipients were dissected and observed for GFP expression under SMZ-1500 stereo-zoom microscope (Nikon Corporation, Tokyo, Japan) fitted with an epi-fluorescence attachment. Images were captured using a DS-5 M camera with Digital sight DS-LI software. Epididymal sperm count was done as described before. The testes were fixed in 4 % paraformaldehyde for 18–20 hours at 4 °C and processed as mentioned previously. Testicular tissue sections were stained with hematoxylin and eosin and were observed under light microscope for evaluating the status of spermatogenesis.

Immunohistochemistry on testicular sections of transplanted recipients were performed according to the method described by us previously [28] using mouse anti-GFP antibody (Clontech, Mountain View, CA, USA) diluted at 1∶250 and incubated overnight at 4 °C. The slides were then exposed to an Alexa Fluor-488-conjugated goat anti-mouse immunoglobulin G (IgG) (Molecular Probes, Eugene, OR, USA) antibody (1∶250 dilution) for 4 hours at room temperature. Sections were analyzed under bright field and ultraviolet illumination (a FITC filter) using a Nikon Eclipse TE2000-S inverted microscope (Nikon Corporation, Tokyo, Japan) attached to a DS-5 M camera assisted by Digital Sight DS-L1 software for capturing the images.

For western blot analysis, total protein was isolated from testes and liver tissues using RIPA (radio-immunoprecipitation assay) lysis buffer [24, 29].Protein concentration was determined using the Bradford assay (Bio-Rad Laboratories, Hemel Hempstead, UK). The samples were boiled in the SDS sample buffer for 5 minutes and were subjected to SDS-PAGE, followed by western blot analysis using primary mouse monoclonal anti-GFP (Santa Cruz Biotechnology, Dallas, TX, USA) antibody at a dilution of 1:2500 and incubated for overnight at 4 °C while the secondary HRP-conjugated anti-mouse IgG antibody (Thermo Fisher Scientific, Waltham, MA, USA; number 31430) at a dilution of 1:5000 for 1 hour at room temperature. β-actin was used as an internal control. For β-actin detection, blot was incubated overnight at 4 °C with rabbit antiserum against β-actin (1:3000) and secondary goat anti-rabbit IgG-HRP antibody (Thermo Fisher Scientific; number 353-1) at a dilution of 1:5000 for 1 hour at room temperature.

### Fertility assessment of GCT mice and analysis of transgenic offspring

Germ cell-transplanted (T-BST-75-GCT) mice were cohabitated with age-matched wild-type females (ratio 1:1) for 10 days after 2 months of busulfan injection to obtain F1 progeny. Since the transplanted SSC were isolated from transgenic mice expressing GFP, pups generated (F1 generation) were analyzed for the presence of GFP by PCR using genomic DNA (gDNA) obtained from their tail tips.

The transgene integration of a few PCR-positive mice were further confirmed by slot blot analysis [30]. A transgene-specific probe was prepared by amplifying a fragment that contained GFP gene (630 bp) using the P1: GACGTAAACGGCCACAAGTT, P2: GGCGGTCACGAACTCCAG primers by PCR. Probes for slot blot analysis were radioactively labeled using αP^32^dCTP by random priming using a High Prime kit from Roche, following the manufacturer’s instructions (Roche Diagnostic GmbH, Mannheim, Germany). About 1 μg of gDNA was blotted on membrane and hybridized with a transgene-specific probe for detection of transgene in the progeny.

Total protein was isolated from the liver of a few slot blot-positive animals and western blot analysis was performed using the procedure described above.

### Unilateral testicular busulfan treatment

Busulfan was also injected at two sites in a single testis of mice to determine its efficacy in Gc depletion.

### Validation of T-BST-75 in other strains of mice

After standardizing the T-BST-75 dose of busulfan in the 1B6SJL hybrid strain of mice, the same dose was validated in the FVB/J strain of mice also.

## Results

### Effect of BST on body weight

**Fig. 1:**
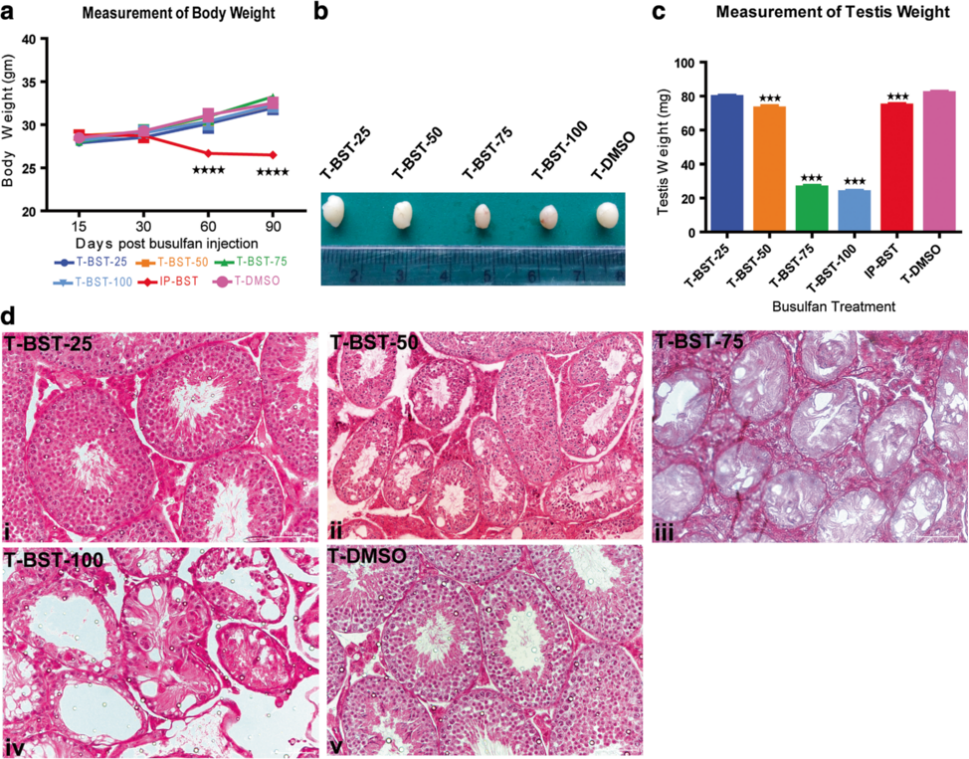
Effect of various doses of testicular busulfan treatment. **a** Changes in body weight of mice injected with various doses of busulfan after 15, 30, 60, and 90 days post BST. Significant (*p* < 0.0001) reduction in the body weight was observed in IP-BST mice (n = 3). **b** Macroscopic observation of testis injected with various doses of busulfan at day 15 post BST. Decrease in testis size with increase in dose of busulfan was observed. Remarkable reduction was found in testis size of T-BST-75 and T-BST-100 mice. **c** Mean testis weight of mice injected with various doses of busulfan. Significant (*p* < 0.0001) reduction was observed in testis weight of T-BST-75 and T-BST-100 mice (n = 3). **d** Hematoxylin and eosin-stained sections of testes, injected with different doses of busulfan at the day 15 post BST. (i) No effect was observed in T-BST-25 mice. (ii) Partial evacuation of seminiferous tubule was observed in T-BST-50 mice. (iii) Maximum depletion of germ cells with intact testicular architecture was observed in T-BST-75 mice. (iv) Maximum depletion of germ cells with disturbed testicular architecture was observed in T-BST-100 mice. Scale bar: 100 μm. T-BST-25, T-BST-50, T-BST-75, T-BST-100 denotes mice injected with 25 μg, 50 μg, 75 μg, and 100 μg of busulfan in each testes respectively. T-DMSO denotes control mice injected with DMSO. IP-BST denotes mice injected intraperitoneally with busulfan

There was no significant difference in the body weight of all the tested groups 15 and 30 days post BST. However, after 60 and 90 days of BST, the body weights were significantly (*p* < 0.0001) reduced in IP-BST group mice in comparison to T-BST and control group mice where the body weight increased with increase in age after busulfan injection (Fig. 1a).

### Effect of BST on testicular parameters

Macroscopic observation of testis at day15 post injection revealed a remarkable reduction in testes size of T-BST-75 and T-BST-100 injected animals as compared to that of control animals (Fig. 1b; Additional file 1: Figure S2). Testicular weight of T-BST-75 and T-BST-100was significantly (p < 0.0001) reduced in comparison to T-BST-25, T-BST-50, IP-BST and control group mice after 15 days of busulfan injection (Fig. 1c).Testicular size and weights proportionally decreased with increase in dose of busulfan injection. Lowest testicular weight was observed in T-BST-75 (26.23 mg) and T-BST-100 (23.33 mg) at day 15 post BST (Fig. 1c).

### Histological morphology of BST mice

**Fig. 2:**
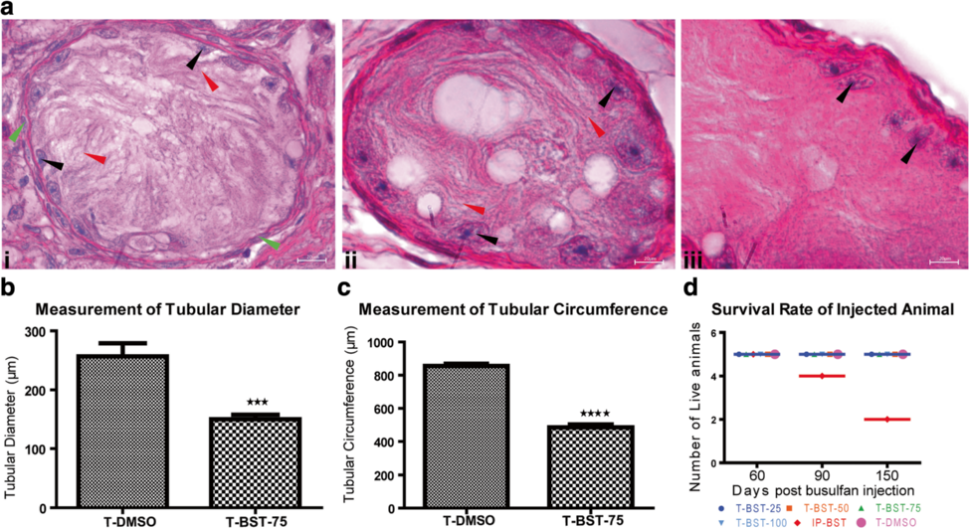
Effect of T-BST-75 on the testicular morphology of mice. **a** A magnified image of hematoxylin and eosin-stained testicular sections of T-BST-75 mice. Maximum depletion of Gc leaving behind only Sertoli cells in the evacuated tubules was observed. *Black arrowhead* marks the nucleus of Sertoli cells, *red arrowhead* marks the cytoplasm of Sertoli cells which was intact in T-BST-75 mice, *green arrowhead* marks the peritubular cells. i, ii and iii are testicular sections from three different mice. Scale bar: 20 μm. **b** Mean tubular diameter (under same magnification) of T-BST-75 mice as compared to T-DMSO control mice. A significant (*p* < 0.05) decrease in tubular diameter was observed in T-BST-75 mice. **c** Mean tubular circumference of T-BST-75 mice as compared to T-DMSO control mice. A significant (*p* < 0.05) decrease in tubular circumference was observed in T-BST-75 mice. **d** Survival rate of mice injected with various doses of busulfan and those treated with vehicle. Survival rate was found to be significantly (*p* < 0.05) reduced in IP-BST mice (n = 5). T-BST-25, T-BST-50, T-BST-75, and T-BST-100 denotes mice injected with 25 μg, 50 μg, 75 μg, and 100 μg of busulfan in each testes respectively. T-DMSO denotes mice injected with DMSO in testes. IP-BST denotes mice injected with busulfan, intraperitoneally

Histological evaluation of testicular sections of T-BST-25 and control mice did not show any effect as seminiferous tubules of such testes remained undisturbed showing all the stages of spermatogenesis in the seminiferous tubules of these mice (Fig. 1d i and v). Testicular sections of T-BST-50 mice revealed partial evacuation, as spermatogenesis was observed in many tubules with presence of many Gc in the innermost and middle layers of the seminiferous tubules (Fig. 1d ii). Seminiferous tubules of T-BST-75 and T-BST-100 mice showed multiple vacuoles, thin-walled seminiferous epithelium and complete absence of sperm (Fig. 1d iii and iv). T-BST-75 had maximum depletion of Gc leaving behind only Sertoli cells in the evacuated tubules (Fig. 2a). T-BST-100 also produced the similar effect as T-BST-75 but T-BST-100 mice showed severe tubular disorganization (Additional file 1: Figure S3). The tubular diameter and circumference was significantly (*p* < 0.05) reduced in T-BST-75 mice (Fig. 2b, c) as the atrophy was very prominent in this group of mice (Additional file 1: Figure S4).The effect of depletion of Gc was observed across the whole testis (Additional file 1: Figure S5).

### Survival rate of testicular busulfan-treated mice

None of the BST mice which were given testicular injections of busulfan (T-BST) died during the entire course of study (100 % survival) in comparison to the IP-BST mice where the mortality rate was 20 % after 90 days (80 % survival) of IP-BST and 60 % after 150 days (40 % survival) of IP-BST respectively. No mortality was observed in DMSO-treated control mice (Fig. 2d).

Since, the mortality rate was high in the IP-BST group and no decrease in testicular weight of the IP-BST group mice was observed at day 15 post BST, they were not analyzed for further studies. Since complete depletion of Gc was not observed during testicular and histological examination of T-BST-25 and T-BST-50 mice, these doses were not used for further studies. T-BST-100 also produced a similar effect as T-BST-75 but T-BST-100 mice showed severe tubular disorganization in comparison to T-BST-75, which never showed any unusual tissue organization, so of the two doses; higher dose, i.e.100 μg, was not used for further analysis. Therefore, the 75 μg dose (T-BST-75) was selected as the most appropriate dose for testicular busulfan injection for maximal depletion of Gc from the testis in 15 days and was used for further studies.

### Effect of BST on peripheral blood cell counts

**Fig. 3:**
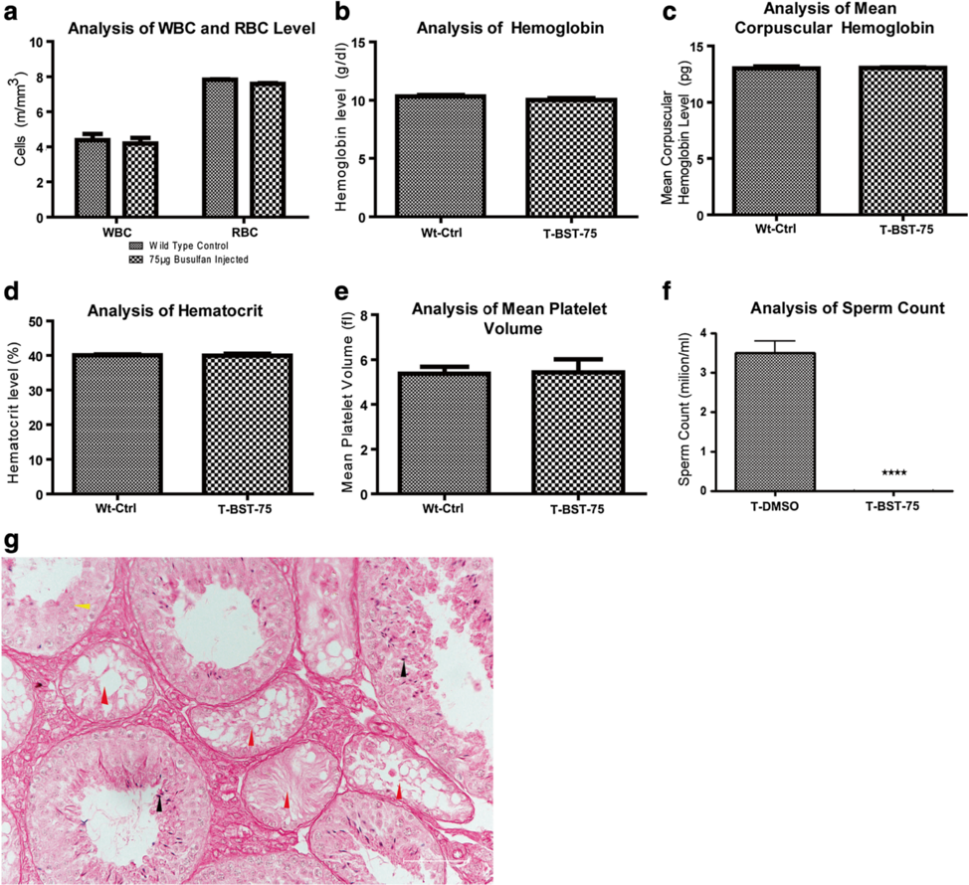
Effect of T-BST-75 on peripheral blood counts and studies of restoration of endogenous spermatogenesis after BST. **a**–**e** Graphs showing changes in levels of white blood cells (WBC), red blood cells (RBC), hemoglobin, platelets, and hematocrit of T-BST-75 mice as compared to wild-type control (Wt-Ctrl) mice. No suppression in peripheral blood counts was observed in T-BST-75 mice. **f** Graph showing caudal epididymal sperm count in T-BST-75 mice 15 days post BST as compared to T-DMSO control mice. No sperm was detected in T-BST-75 mice. (n = 3, *p* < 0.005). **g** Hematoxylin and eosin-stained testicular sections of T-BST-75 mice dissected 2 months after BST. Only a few of the non-spermatogenic tubules showed recovery of spermatogenesis at 2 months after BST. *Black arrowhead* marks the sperm, *red arrowhead* marks the tubules, in which spermatogenesis has not yet initiated, *green arrowhead* marks the proliferating Gc. All data were recorded 15 days post BST unless mentioned otherwise. T-BST-75 denotes mice injected with 75 μg of busulfan in each testes, T-DMSO denotes mice injected with DMSO in testes

No suppression in peripheral blood counts was observed in T-BST-75 mice in comparison to wild-type control mice (Wt-ctrl). The white blood cell (WBC) counts did not show any significant (*p* > 0.05) difference in both the groups (Fig. 3a). Red blood cell (RBC) counts and hemoglobin were also similar and did not show any significant (*p* > 0.05) difference in both the groups (Fig. 3a, b). Mean corpuscular hemoglobin and hematocrit levels were also significantly similar (*p* > 0.05) in both groups (Fig. 3c, d). There was no significant (*p* > 0.05) difference in the levels of platelets too (Fig. 3e). None of the T-BST-75 mice required bone marrow transplantation as hematopoietic suppression was not observed in any of the mice.

### Effect of BST on sperm count and litter size

Fifteen days post busulfan injection, no sperm was detected in T-BST-75 mice in comparison to T-DMSO mice where the sperm count was 3.5 million/ml (Fig. 3f). T-BST-75 mice were found to be infertile as they failed to produce any offspring when they were cohabitated with age-matched females, 15 days post BST.

### Restoration of endogenous spermatogenesis after BST

Restoration of endogenous spermatogenesis without SSCT was initiated in few tubules 2 months after busulfan injection (Fig. 3g).

### Restoration of fertility in T-BST-75 infertile recipient mice by germ cell transplantation of in vitro expanded transgenic SSC

**Fig. 4:**
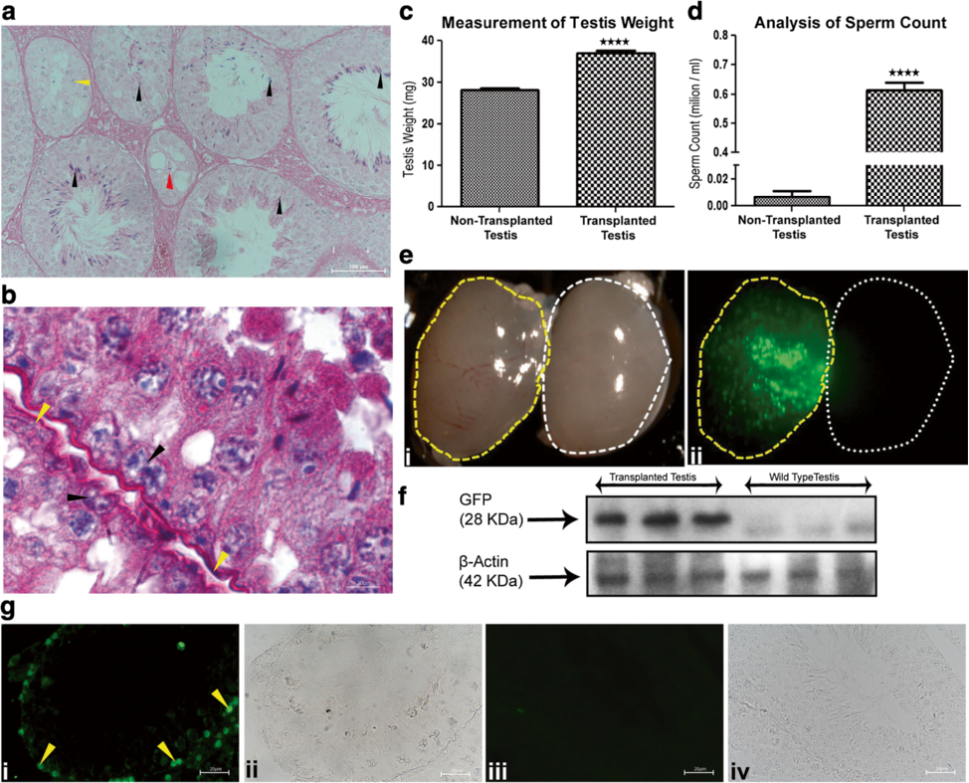
Restoration of fertility in T-BST-75 mice after GCT. **a** Hematoxylin and eosin-stained testicular sections of T-BST-75-GCT mice. Complete stages of spermatogenesis were observed in many tubules. Several tubules only showed initial stages of spermatogenesis. Few tubules were also seen in which spermatogenesis was not started. *Black arrowhead* marks the sperm, *green arrowhead* marks the proliferating Gc. Scale bar: 100 μm. **b** A magnified image of hematoxylin and eosin-stained testicular sections of T-BST-75-GCT mice. *Black arrowhead* shows two Gc which were harbored in a single evacuated space, *yellow arrowhead* marks the basement membrane. Scale bar: 100 μm. **c** Mean testis weight of T-BST-75-GCT mice as compared to non-transplanted control mice. A significant (*p* < 0.0001) increase in testes weight was observed in the transplanted testes (n = 10). **d** Mean sperm count of T-BST-75-GCT mice as compared to non-transplanted control mice. A significant (*p* < 0.0001) increase in sperm count was observed in the transplanted testes (n = 10). **e** In vivo GFP expression in T-BST-75-GCT testes as compared to non-transplanted control testes 2 months after GCT. GFP expression was observed in transplanted testes. i Image under bright field stereo-zoom microscope. ii Image under UV with FITC filter. *Yellow dotted line* marks the transplanted testis. *White dotted line* marks the non-transplanted testis. **f** Western blot of total protein isolated from T-BST-75-GCT testes as compared to wild-type testis. Transplanted testis showed presence of 28 KDa protein corresponding to GFP. β-actin was used as internal control. **g** Immunostaining showing GFP expression in the Gc of seminiferous tubules of T-BST-75-GCT testis. GFP expression was observed specifically in Gc of the testis. i and ii shows testicular sections of GCT testis. iii and iv shows testicular sections of non-transplanted testes. *Yellow arrowhead* shows GFP expression in the Gc. Scale bar: 100 μm. All data were recorded 2 months after GCT

Donor SSC from C57BL/6-Tg(UBC-GFP)30Scha/J mice carrying GFP transgene, sorted through FACS (Additional file 1: Figure S6) and expanded in stem cell-specific media (Additional file 1: Figure S7 and S8) were transplanted in the evacuated T-BST-75 testis of F1BL6SJL recipient mice (Additional file 1: Figure S9). The GCT mice were housed for 2 months for colonization of transplanted SSC (Fig. 4a, b). Two months post transplantation, the testicular weight of GCT mice had increased significantly (*p* < 0.0001, 37 mg) as compared to non-transplanted T-BST-75 control mice (28 mg) (Fig. 4c). Epididymal sperm count from GCT mice was also found to be significantly (*p* < 0.0001) higher (0.61 million/ml) than that of non-transplanted T-BST-75 mice (0.007 million/ml) at 2 months post GCT (Fig. 4d). Transplanted testes also showed in vivo GFP expression under stereo-zoom microscope with a FITC filter upon excitation with UV, which was absent in non-transplanted testes (Fig. 4e). Western blot analysis of total protein from GCT testes showed the presence of 28 KDa protein corresponding to GFP, which reconfirmed colonization of GFP-expressing donor SSC (Fig. 4f). Immunostaining of GCT testes showed GFP expression confirming colonization of transplanted donor SSC (Fig. 4g). GFP expression was observed in the transplanted testes only, but not in the non-transplanted T-BST-75 mice testes.

### Fertility assessment of GCT mice at 2.5 months post BST

**Fig. 5:**
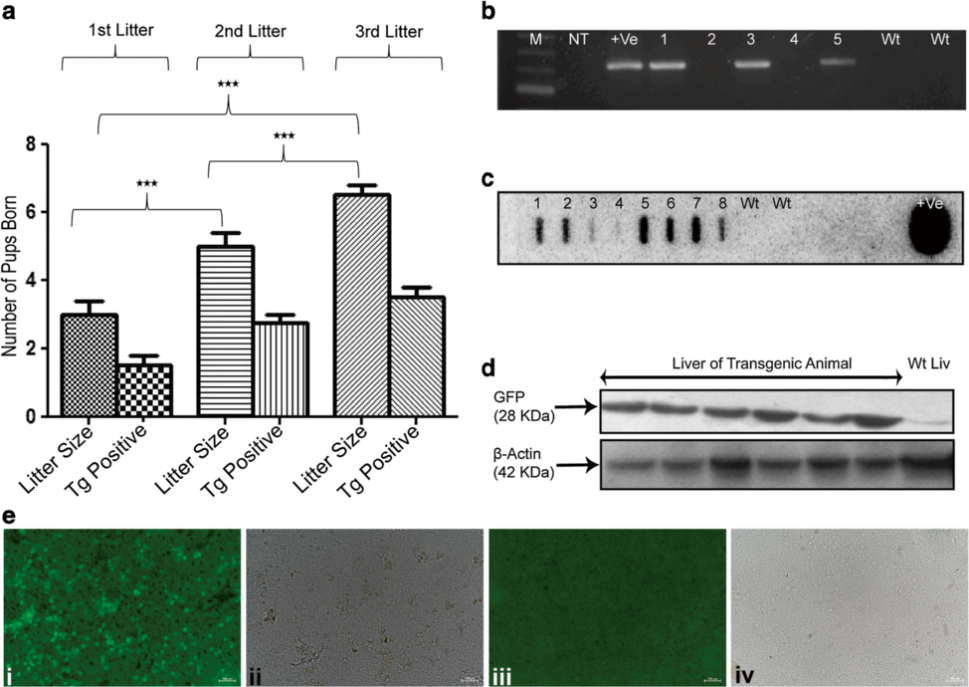
Generation and analysis of transgenic offspring. **a** Litter size and number of transgenic positive pups in F1 generation of each litter sired by T-BST-75-GCT mice. A significant (*p* < 0.0001) increase in litter size of subsequent F1 generations was observed with same T-BST-75-GCT males (n = 10). **b** PCR screening for transgene positive pups in a litter sired by T-BST-75-GCT mice. Lanes 1, 3, and 5 shows the presence of transgene in gDNA of pups. M denotes NEB 1 kb DNA ladder; NT denotes no template control, +Ve denotes plasmid positive, 1–5 denotes lanes with gDNA of pups; Wt denotes gDNA of wild-type mice. **c** Slot blot for confirmation of transgene in PCR positive pups. Six out of eight (slot 1–8) showed strong positive hybridization for transgene. Wt denotes gDNA of wild-type mice, +Ve denotes plasmid positive. **d** Western blot of total protein isolated from liver of transgene positive pups. All the transgenic pups showed GFP expression in the liver samples as compared to wild-type. β-actin was used as internal control. **e** Immunostaining showing GFP expression in the liver sections of transgenic pups. i and ii show liver sections of transgenic pups. iii and iv show liver sections of wild-type mice. Scale bar: 100 μm

Germ cell transplanted (T-BST-75-GCT) mice were cohabitated with age-matched wild-type females for 10 days after 2 months of busulfan injection to obtain F1 progeny pups. There was no delay in pregnancy of female mice which were mated with T-BST-75-GCT mice and pups were obtained between 19–21 days of gestation period with average litter size of three pups per mouse (Fig. 5a). Out of 34 GCT males set for mating (1:1 scheme, total 34 females) at 2.5 months post BST, 24 males sired 69 pups. The average litter size of T-BST-75-GCT mice, which was three initially at 2.5 months post BST, gradually increased to seven after 4 months of BST (Fig. 5a). No pups were obtained in non-transplanted T-BST-75 control (T-BST-75-NT) mice, when set for mating after 2.5 months of BST but pups were obtained after 4 months of BST treatment (data not shown). The average litter size of T-BST-75-NT mice was less (two pups) in comparison to T-BST-75-GCT mice, which showed an average litter size of seven after 4 months of busulfan injection.

### Generation and analysis of transgenic offspring

Sixty-nine pups born from T-BST-75-GCT mice were analyzed for the presence of transgene by PCR and slot blot analysis. The presence of 633 bp band in PCR analysis showed the amplification of GFP transgene (Fig. 5b). Slot blot analysis of PCR-positive pups confirmed the presence of transgene (Fig. 5c). Western blot analysis of the protein isolated from the liver of slot blot-positive pups showed the presence of 28 KDa protein corresponding to GFP, which reconfirmed that the offspring of GCT mice had originated from transgenic donor SSC (Fig. 5d). Immunostaining of liver sections of slot blot-positive pups showed GFP expression confirming the presence of transgene (Fig. 5e). Out of 69 pups analyzed, 39 pups were found to express GFP (56 % transgenic efficiency). All 24 females which were impregnated by T-BST-75-GCT male mice sired donor-derived transgenic pups with average transgenic efficiency of 56 %.

### Unilateral testicular treatment for depleting germ cells in single testis

We also experimented to evacuate single testis of mice through direct testicular injection of busulfan. Seventy-five micrograms of busulfan dose was administered in single testis of a mouse keeping the contralateral testis as control. Macroscopic observation after 15 days of BST revealed that the T-BST-75 testes were smaller in size as compared to that of non-injected contralateral control testes (Additional file 1: Figure S10). Histological observation of T-BST-75 testes showed almost complete depletion of Gc leaving behind only Sertoli cells in the evacuated tubules. The effect of busulfan was confined to a single testis only. This effect was similar to the effect observed by giving busulfan injection in both testes. The contralateral control testes showed no cytotoxic effect of busulfan as the testis was intact and organized, showing all stages of Gc.

### Validation of T-BST-75 in other strains of mice

After standardizing the T-BST-75 dose of busulfan in F1B6SJL hybrid strain of mice, we validated the same dose in FVB/J strain of mice. A 75 μg dose was found to be equally effective in the FVB strain of mice and was successful in depleting the Gc and evacuating the seminiferous tubules in 15 days after busulfan injection (Additional file 1: Figure S11).

## Discussion

We have established an efficient method of testicular busulfan injection that required lower dose of busulfan to prepare recipients for GCT while achieving the objective of maximum Gc depletion without any cytotoxic effect in other organs. Our method is very efficient in terms of offspring generated (56 %) from SSC-transplanted males; rapid in terms of time required to evacuate testis using lowest effective dose of busulfan (75 μg) without any systemic ill effect. The success rate of our method for obtaining donor-derived offspring is very high (71 %) as compared to such methods reported recently by other group (5.5 %) as only 1 out of 18 Gc-transplanted mice could generate offspring from transplanted SSC in their study [21].

For this study, we have used F1B6SJL strain of mice for initial standardization of busulfan dosage for direct administration into the testes to prepare recipient mice prior to SSCT. We tested several doses of testicular busulfan (25 μg, 50 μg, 75 μg, and 100 μg) and found that the 75 μg dose provided desirable results as compared to a recently reported method [21] where 6 mg/kg^-1^dose (approximately 180 μg busulfan per testis) was selected as an appropriate dose for recipient preparation. This is 2.4 times more than ours (75 μg/testis).

There have been several reports which showed that i.p. injections of busulfan can cause systemic toxicity including severe bone marrow depression and/or death of animals [4, 12, 31]. In our study, no mortality was observed in testicular BST mice while i.p. injection of busulfan caused 60 % mortality. This confirmed that direct administration of busulfan in testis at a lower dose did not induce any cytotoxic effect on the hematopoietic system.

The body weight of T-BST and control group mice increased with age unlike the IP-BST group where the body weight remained low as compared to age-matched untreated animals.

Testicular size and weight proportionally decreased with increase in dose of busulfan injection. Lowest testicular weight was found in T-BST-75 and T-BST-100 at day 15 post BST. This was followed by a significant decrease in the tubular diameter. The prominent decrease in the diameter of the seminiferous tubule might have occurred due to depletion of the luminal contents. This was supported by histological observations like depletion of Gc, presence of multiple vacuoles, absence of sperm and presence of hollow cavities in the seminiferous tubules of T-BST. Maximum depletion of Gc was observed in T-BST-75 and T-BST-100 group mice 15 days post BST but T-BST-100 mice showed severe tubular disorganization which was not discernible in T-BST-75 mice. The depletion of Gc with our method was 7 days earlier than the one reported recently using 6 mg/ kg^-1^ testicular busulfan where maximum depletion was noticed at day 21 after BST [20, 21]. The major difference between our procedures is the use of two diagonally opposite sites for testicular injection of busulfan by us and from a single site through the tail end of testis by them [20, 21]. We injected busulfan from two sites (10 μl at each site) through both ends (the upper as well as lower end of the testis) covering the whole testicular area, thereby the effect of busulfan was more pronounced. In a previously reported study, injection was given from the testicular tail side along the long axis of the testis. In the process of travelling from one end to another, busulfan may get diluted by testicular lymph nodes thereby requiring higher doses and duration for the desirable effect.

None of the T-BST-75 mice required bone marrow transplantations as hematopoietic suppression was not observed in any of the mice, as opposed to conventional way of busulfan administration (i.p.) where whole body gets exposed to this cytotoxic drug causing depletion of various stem cell populations in other body organs too.

Restoration of endogenous spermatogenesis without SSCT was observed in few seminiferous tubules at 2 months after busulfan injection and gradually increased with time showing maximum restoration by 4 months. Since non-spermatogenic state of the BST-T-75 mice was attained at 15 days post BST, transplantation experiments may be performed 25 days earlier in contrast to the conventional method (i.p.). In the conventional method, Gc depletion and removal of sloughed off Gc occur by 6 weeks after busulfan injection, after which the transplantation experiments are performed [32]. However, by our method, GCT can be performed 25 days earlier. Also with our method, the depleted state of the testis is sustained for a longer period (>1 month) thereby increasing the window for undertaking transplantation studies.

Donor-derived germ cell colonies in testes of T-BST-75 recipients showing GFP expression were observed within 2 months post transplantation, indicating successful GCT. In all, 24 out of 34 such males could successfully impregnate females within 10 days of cohabitation. However, males treated with busulfan but not transplanted with SSC (n = 3) could impregnate females only at 4.5 months after BST. Based on this, it may be assumed that out of 34 T-BST-75 mice, attempted for GCT, 24 were transplanted successfully and efficiently because of which they (71 % success rate) sired pups at about 2.5–3 months after BST. Out of 69 offspring analyzed, 39 were found to be transgene positive (56 % transgenic efficiency). This transgenic efficiency rate with our method of BST is very high in comparison to 1.2 % shown previously as only 4 out of 335 pups were found to be transgene positive originated from donor SSC which were transplanted exogenously [21] and all these offspring were born from only 1 of the 18 recipient mice, with success rate of 5.5 % only [21]. The reason for their low efficiency might reside in remarkably reduced cytoplasm of Sertoli cells observed in the cross section of testes due to busulfan treatment, at doses higher than ours (120–180 μg as compared to 75 μg used by us). They also have transplanted SSC at a time when the testis was not sufficiently depleted of Gc, reducing their efficiency. Our method is hence, superior to the previously suggested method.

Unilateral testicular busulfan treatment for depleting Gc in single testis was quite effective by our method without any nonspecific cytotoxic effect of busulfan on any other organ including contralateral testis. While in the conventional i.p. route of busulfan administration, whole body get exposed to this cytotoxic drug, which causes the depletion of other stem cell population in the body, this unilateral testicular treatment may be advantageous for many studies where one testis can be used for experimental purpose and contralateral testis may be used as an internal control.

Our method of recipient preparation using the F1B6SJL strain of mice, when checked in other strains of mice was found to be equally effective and successful in evacuating the seminiferous tubules in 15 days after busulfan injection. We also suggest that species-wise doses of busulfan could be selected for better effect at the lowest dose, causing damage to SSC but sparing other cell types of the testes like Sertoli cells or Leydig cells, which help restoration of spermatogenesis. It has also been reported in the past that higher doses of busulfan also affect the Sertoli cells and Leydig cells [33].

Here, we have established an efficient method of Gc depletion by testicular busulfan injection using a lower dose of busulfan to prepare GCD recipients while achieving the objective of maximum Gc depletion without any cytotoxic effect to other organs. The success rate by our method is 56 % which is severalfold more as compared to 1.2 % shown earlier [21]. Our method is fast, efficient, and ethically superior as it will utilize less animals to generate more information.

## Conclusions

We have established an efficient method of generating a germ cell-depleted animal model by using a lower dose of busulfan, injected through two different and diagonally opposite sites in the testis, which allows efficient colonization of transplanted SSC and leads to a remarkably higher proportion of donor-derived offspring generation.

## Additional file

Additional file 1: Supplementary Information. (PDF 2480 kb)

## Abbreviations

BST: Busulfan treatment
Gc: Germ cells
GCD: Germ cell depletion recipients
GCT: Germ cell transplantation
gDNA: Genomic DNA
i.p.: Intraperitoneal
IP-BST: Intraperitoneal busulfan treatment
RBC: Red blood cells
SSC: Spermatogonial stem cells
SSCT: Spermatogonial stem cell transplantation
T-BST: Testicular busulfan treatment
WBC: White blood cells
