# Supplementary material for: An efficient method for generating a germ cell depleted animal model for studies related to spermatogonial stem cell transplantation
Source: Stem Cell Res Ther. 2016 Sep 22;7:142. doi: 10.1186/s13287-016-0405-1 (PMC5032248; doi:10.1186/s13287-016-0405-1)
Supplement: Additional file 1: — Supplementary Information. (PDF 2480 kb) [file 13287_2016_405_MOESM1_ESM.pdf]

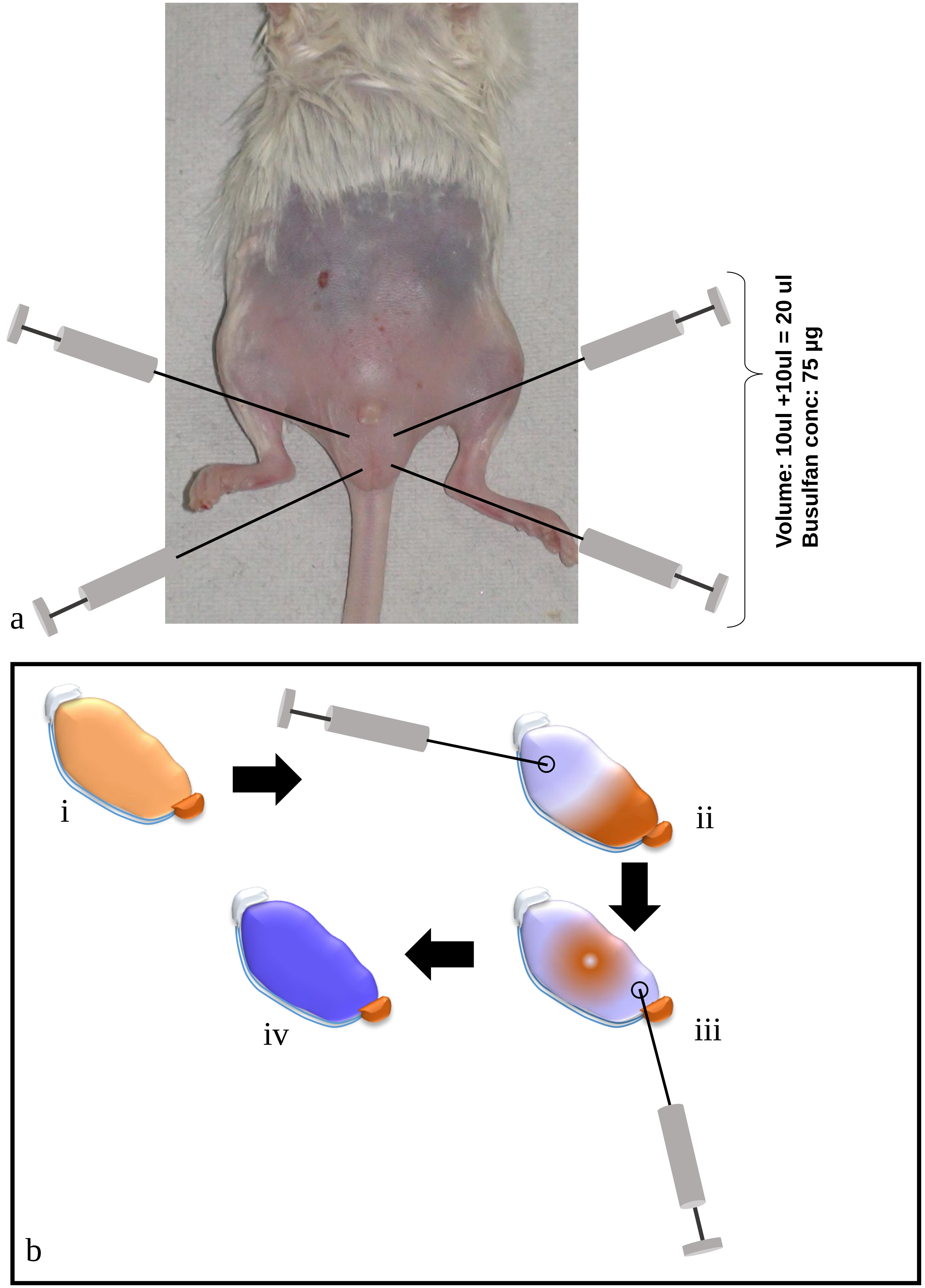

Fig. S1. Schematic representation of the direct testicular injection of busulfan at two different and diagonally opposite site of the testis (10µl at each site, 20µl/testis) in mice.

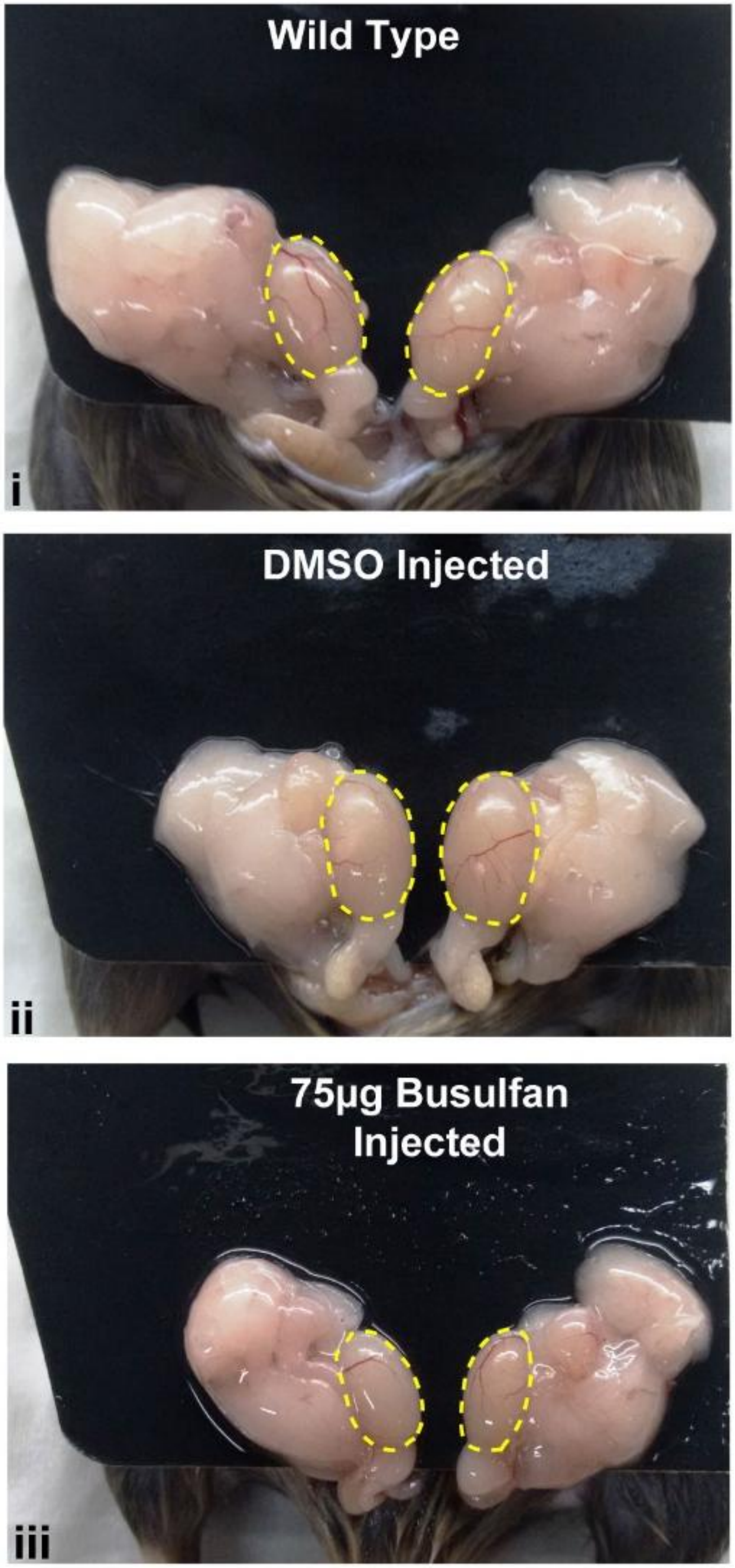

Fig. S2. Macroscopic observation of T-BST-75 testis 15 days after busulfan injection. T-BST-75 mice showed significant reduction in testis size after 15 days of treatment.

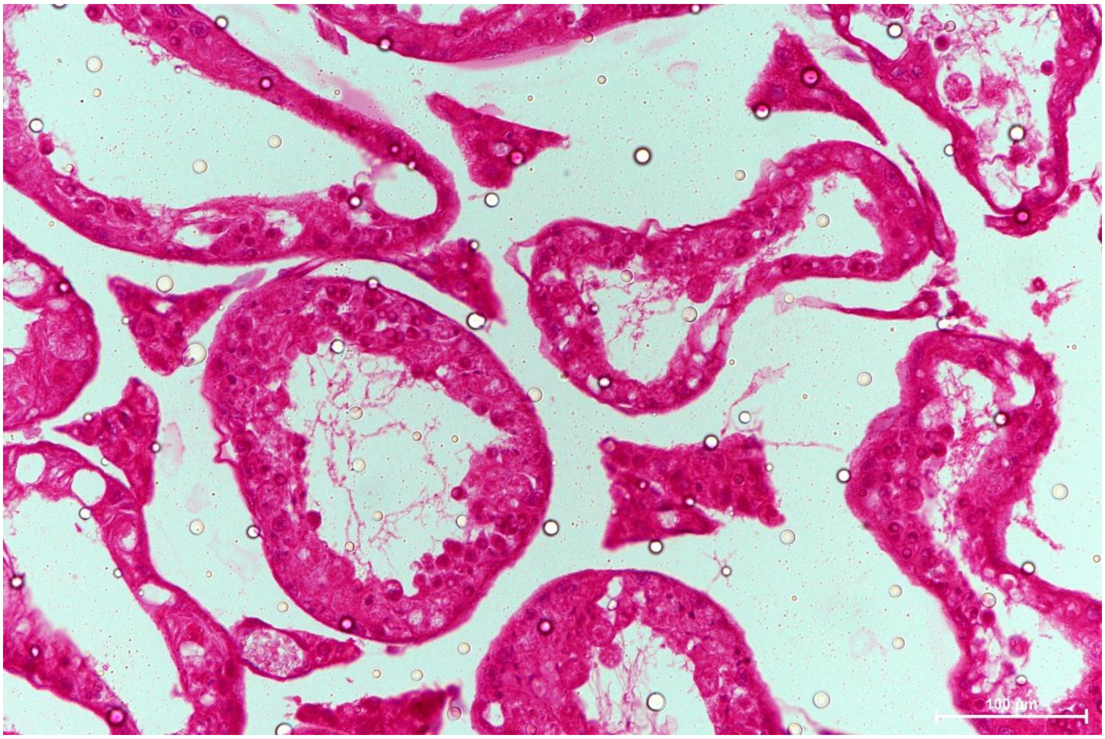

Fig. S3 : Hematoxylin and eosin stained section of T-BST-100. Severe tubular disorganization was observed. Scale bar 100μm.

T-BST-75

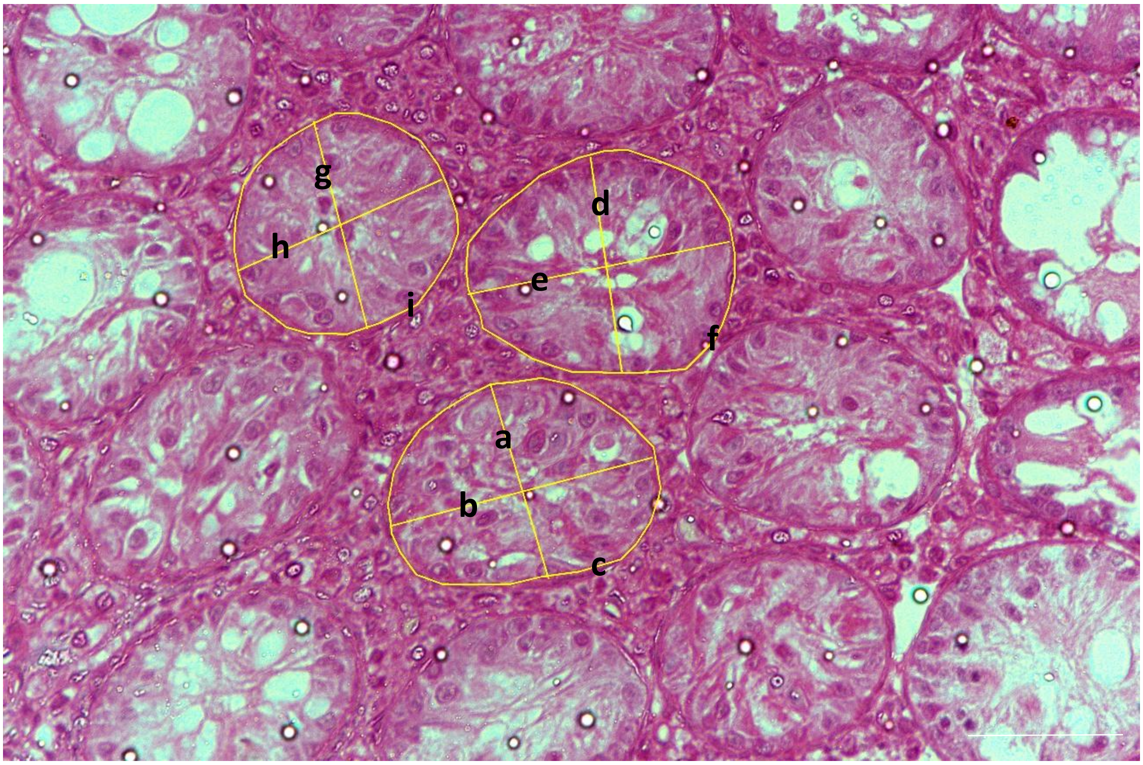

Tubule Diameter
a:130.37μm
b:173.95μm
d:140.16μm
e:173.01μm
g:138.53μm
h:144.76μm

Tubule Circumference
c:497.62μm
f:507.91μm
i:451.28μm

T-DMSO

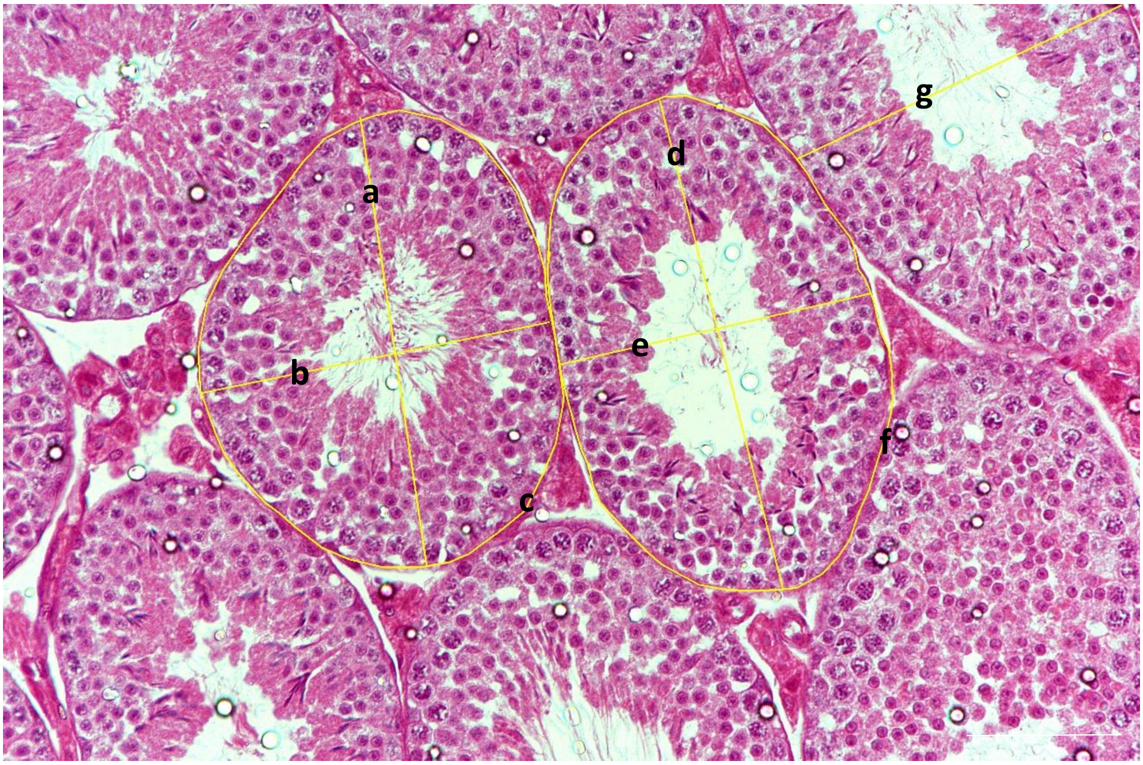

Tubule Diameter
a:292.32μm
b:230.12μm
d:325.34μm
e:206.35μm
g:232.00μm

Tubule Circumference
c:882.43μm
f:866.92μm

Fig.S4: Hematoxylin and eosin stained testicular sections of T-BST-75 and T-DMSO mice showing measurement of tubular diameter and circumference. The recorded data is presented besides the respective image. Scale Bar: 100μm.

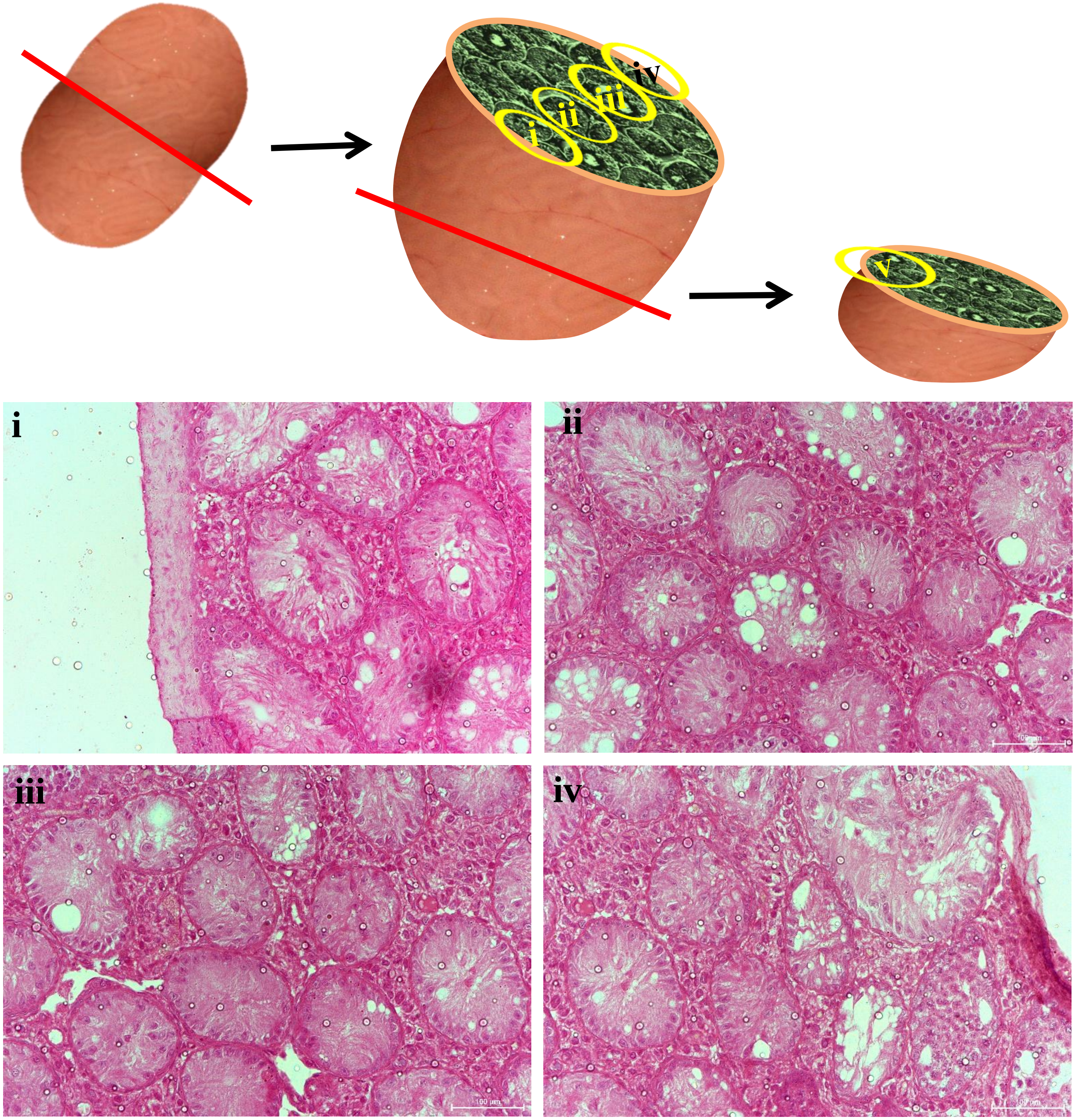

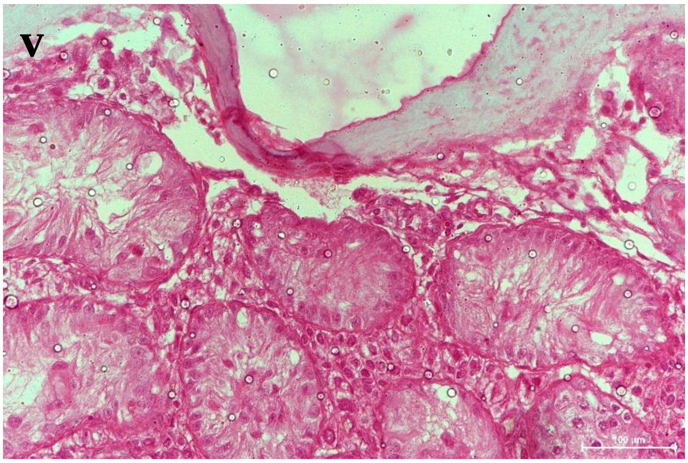

Fig.S5: Hematoxylin and eosin stained testicular sections of T-BST-75 mice.
i, ii, iii, iv, v: Several images were captured moving across the surface of the section (with constant z axis) starting from the one end to the another covering the whole diameter of the testis (as described in the cartoon diagram above). The similar effect of depletion of Gc was observed across the whole testis.

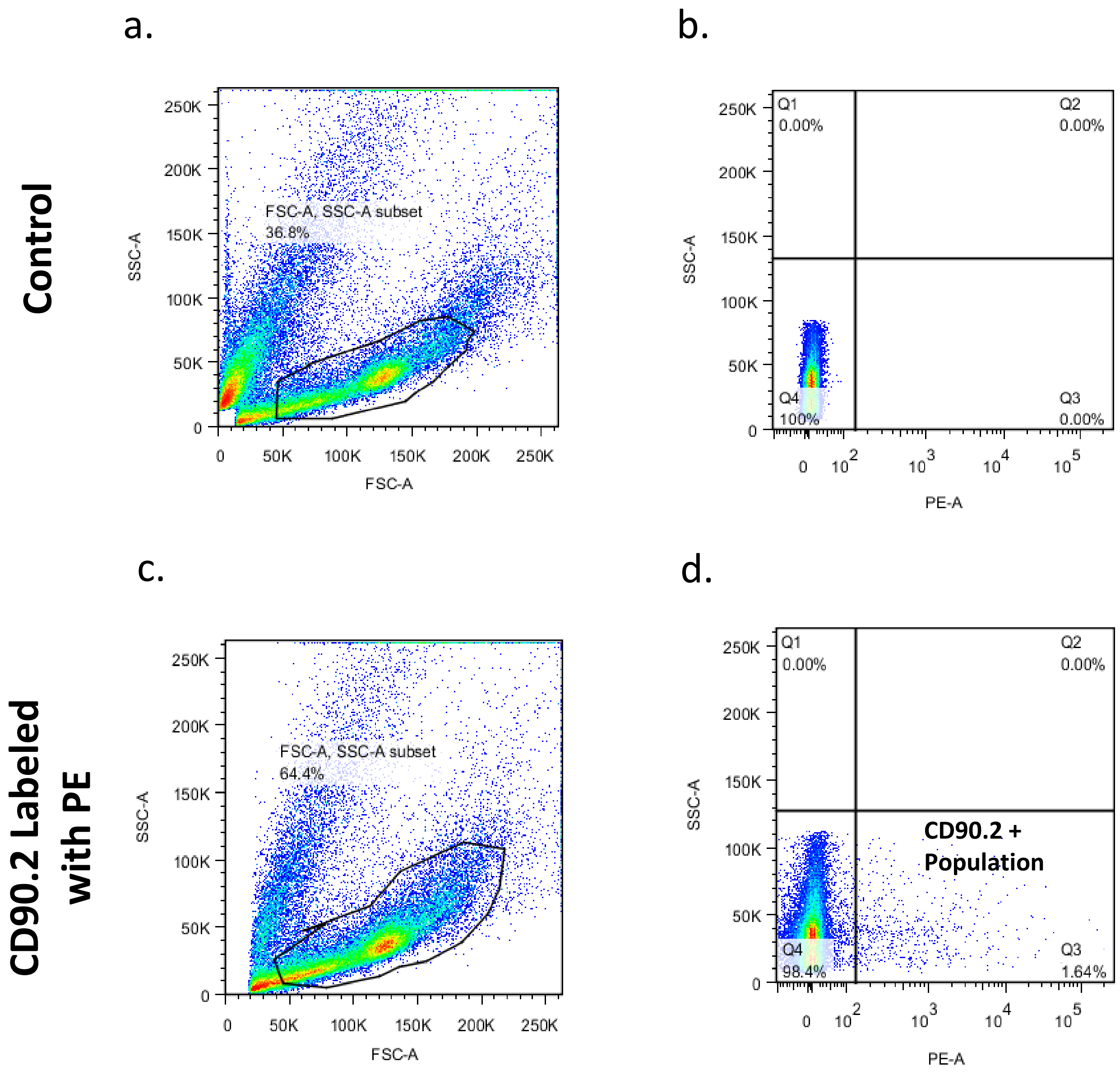

Fig. S6: Enrichment of the SSC from total testicular germ cell population by FACS using CD90.2 spermatogonial cell surface marker.

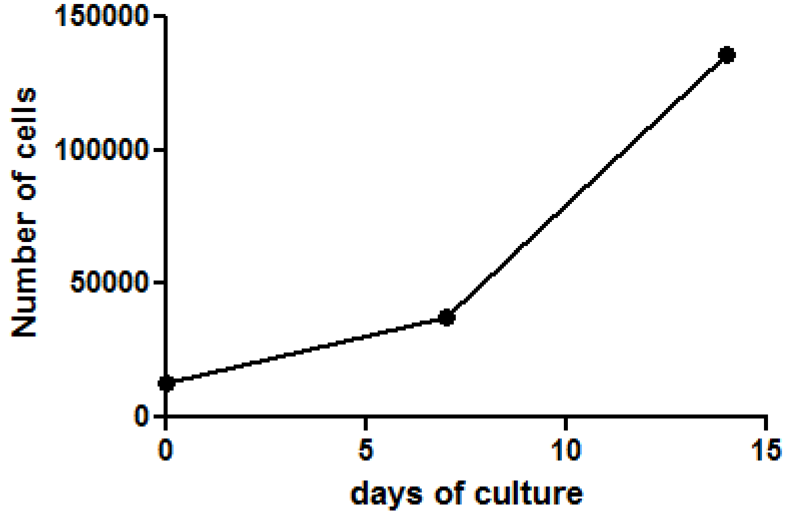

Fig.S7: Proliferation rate of SSC isolated from adult testis.

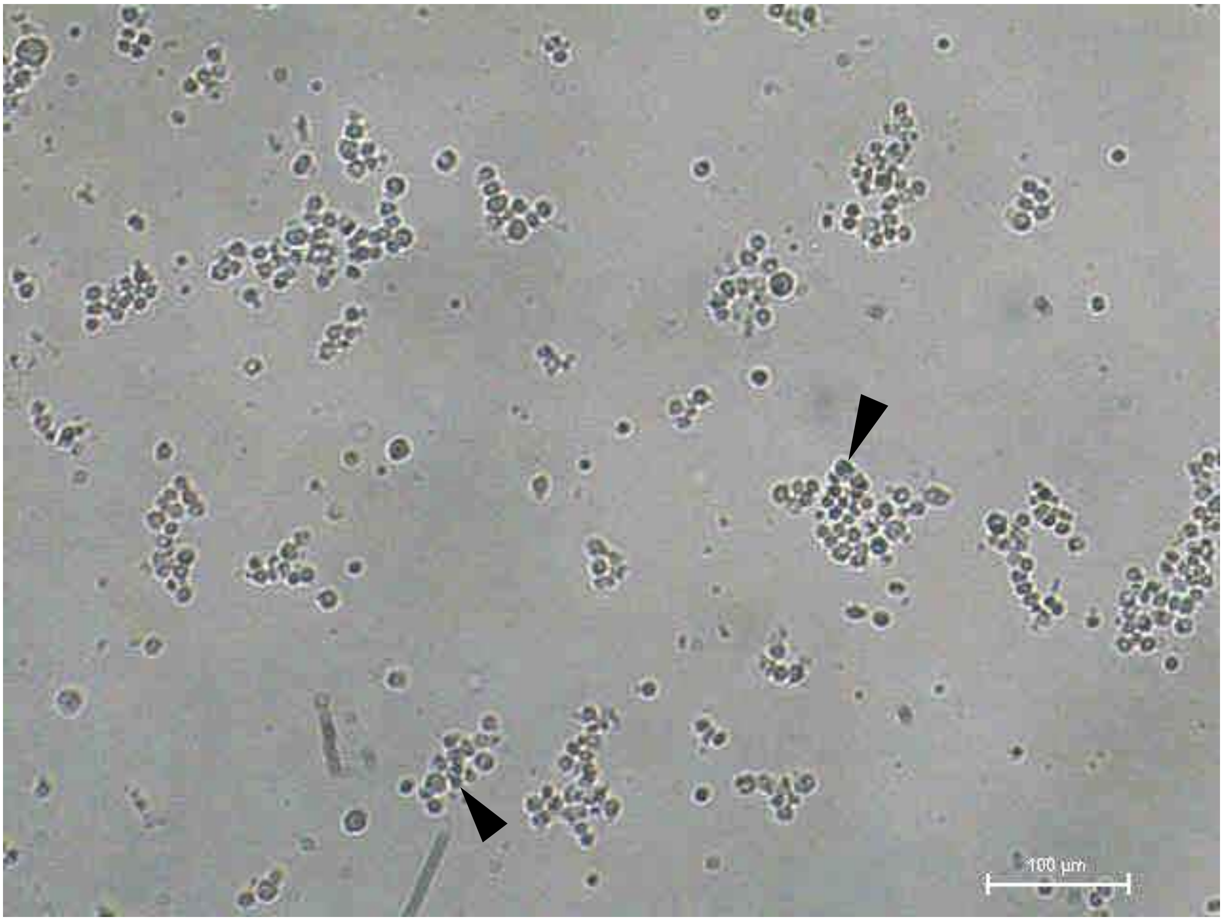

Fig. S8: FACS sorted SSC in culture. Black Arrow head represents grape like colonies of SSC. Scale bar: 100µm

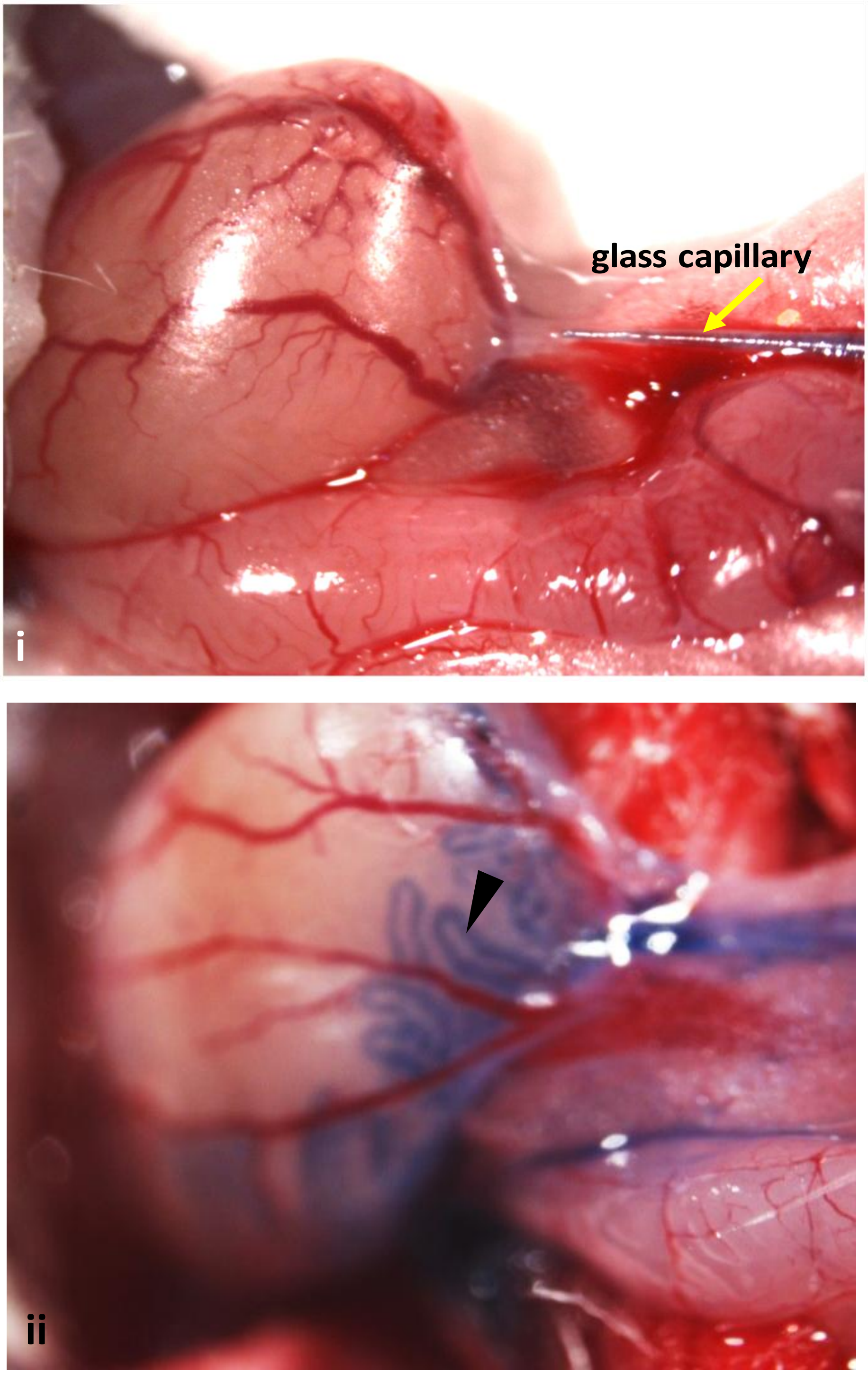

Fig. S9: Germ Cell Transplantation into seminiferous tubules of mice through efferent duct. **i.** Insertion of glass capillary into the efferent duct. **ii.** Transplantation of Gc into seminiferous tubules of mice. Blue colour of trypan blue can be seen in the seminiferous tubules (black arrow head).

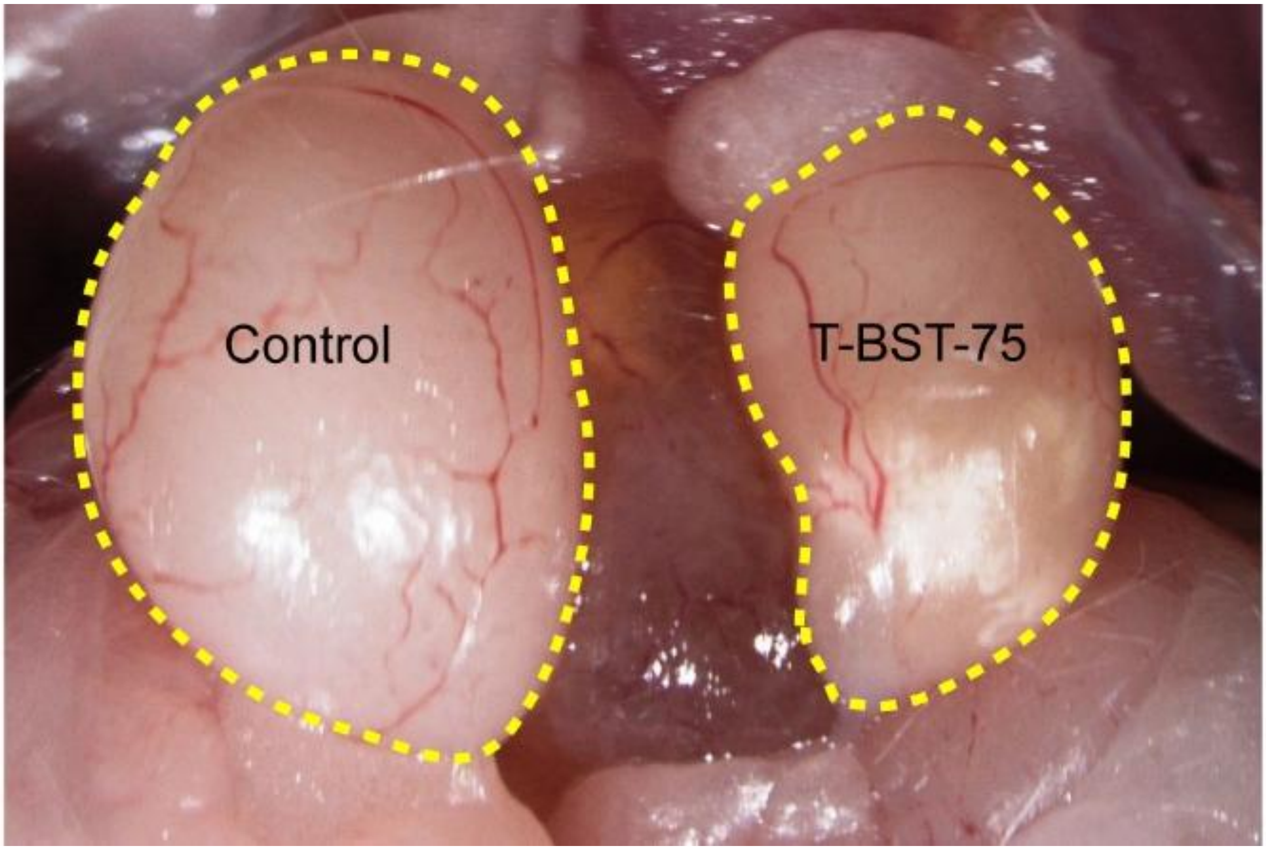

Fig.S10: Unilateral testicular busulfan treatment for depleting Gc in single testis. Only T-BST-75 testis showed reduction in size in comparison to contralateral control testis.

T-BST-75 **Wild Type Control**

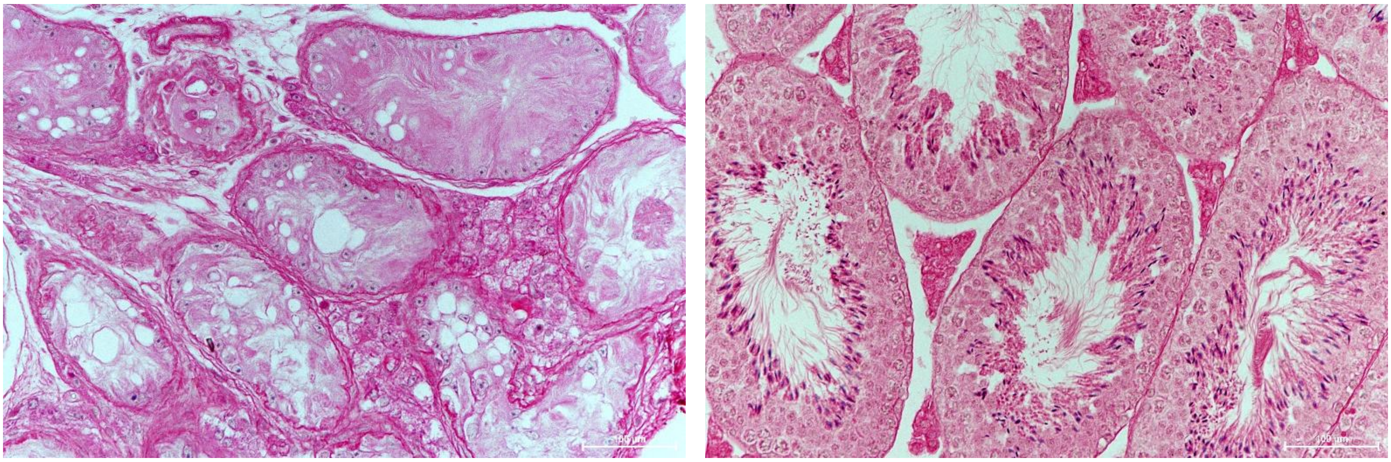

Fig.S11 : Validation of T-BST-75 dose in FVB strain of mice. Hematoxylin and eosin stained testicular sections of T-BST-75 and T-DMSO treated FVB mice. 75µg dose was found to be equally effective in depleting Gc 15 days post BST.
